# Supplementary material for: Differential Alterations in Resting State Functional Connectivity Associated with Depressive Symptoms and Early Life Adversity
Source: Brain Sci. 2021 May 2;11(5):591. doi: 10.3390/brainsci11050591 (PMC8147478; doi:10.3390/brainsci11050591)
Supplement: Supplementary file 1 [file brainsci-11-00591-s001.zip › brainsci-1209800-supplementary.pdf]

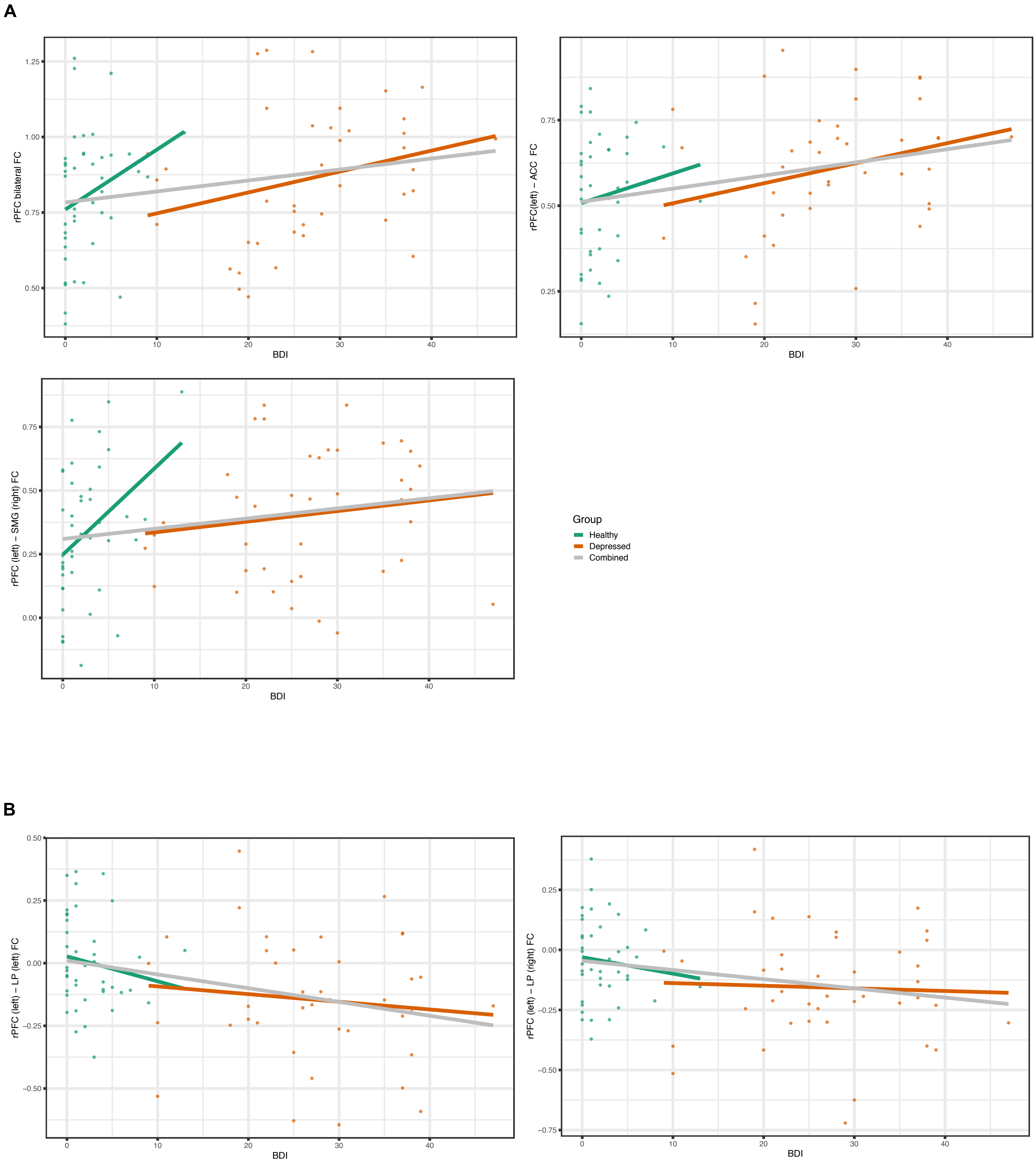
A
rPFC bilateral FC
BDI
rPFC(left) – ACC FC
BDI
rPFC (left) – SMG (right) FC
BDI
Group
Healthy
Depressed
Combined
B
rPFC (left) – LP (left) FC
BDI
rPFC (left) – LP (right) FC
BDI

C

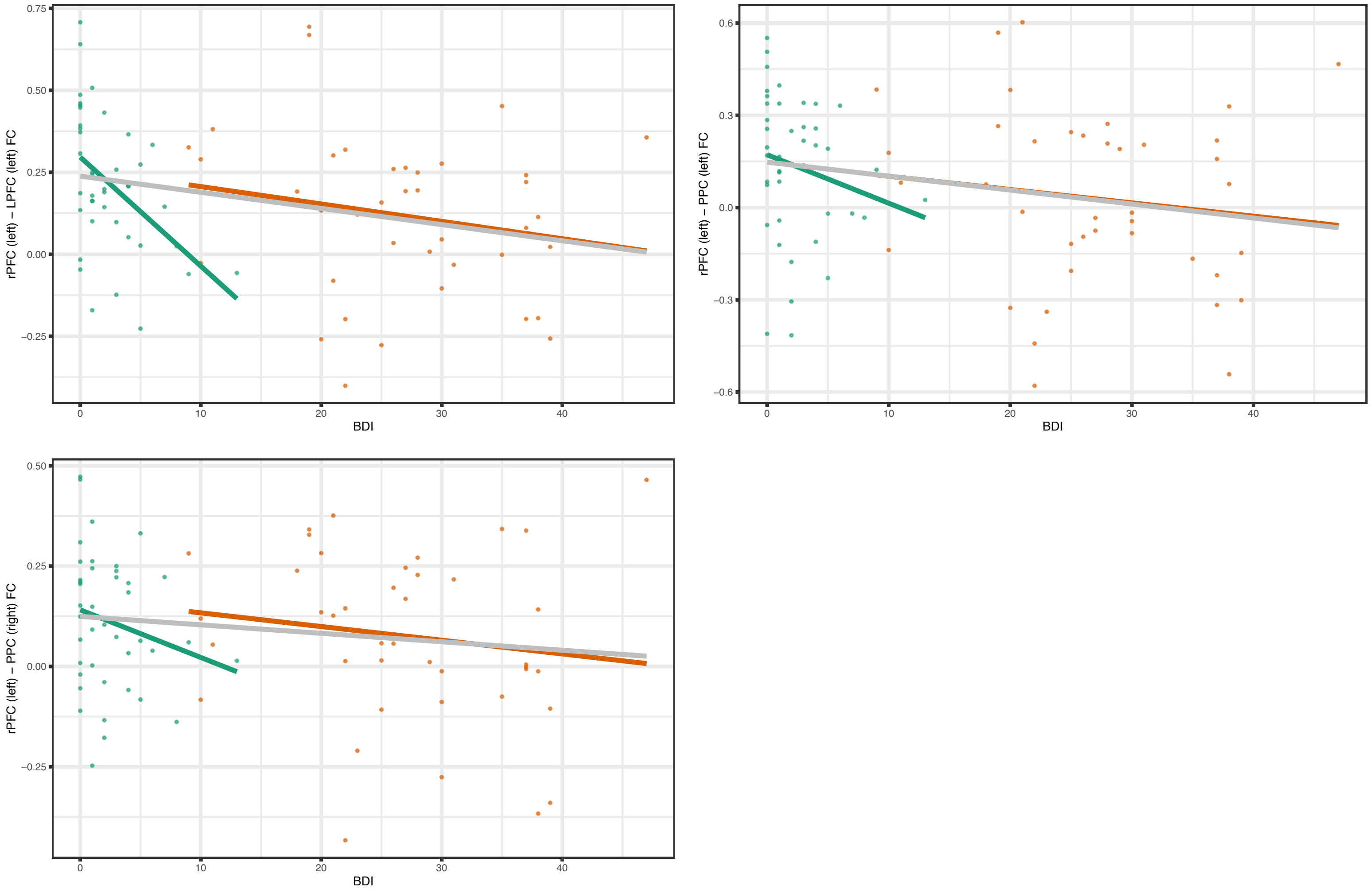

Figure S1. Associations between BDI-II and ROI-to-ROI functional connectivity (FC) for the depressed group, the healthy group, and combined, sorted by within- and between-network FC. A. Associations between BDI-II and FC within the Salience Network. B. Associations between BDI-II and FC of the Salience with the Default Mode Network. C. Associations between BDI-II and FC of the Salience with the Central Executive Network. ROI = region of interest, rPFC = rostral prefrontal cortex, ACC = anterior cingulate cortex, SMG = supramarginal gyrus, LP = lateral parietal, LPFC = lateral prefrontal cortex, PPC = posterior parietal cortex.

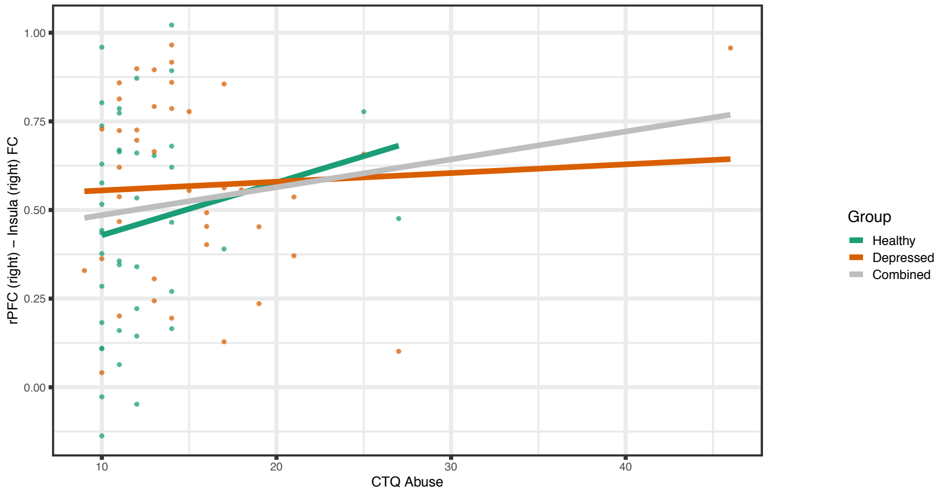

Figure S2. Association between CTQ abuse and functional connectivity (FC) of the right rostral prefrontal cortex (rPFC) with the right Insula for the depressed group, the healthy group, and combined.

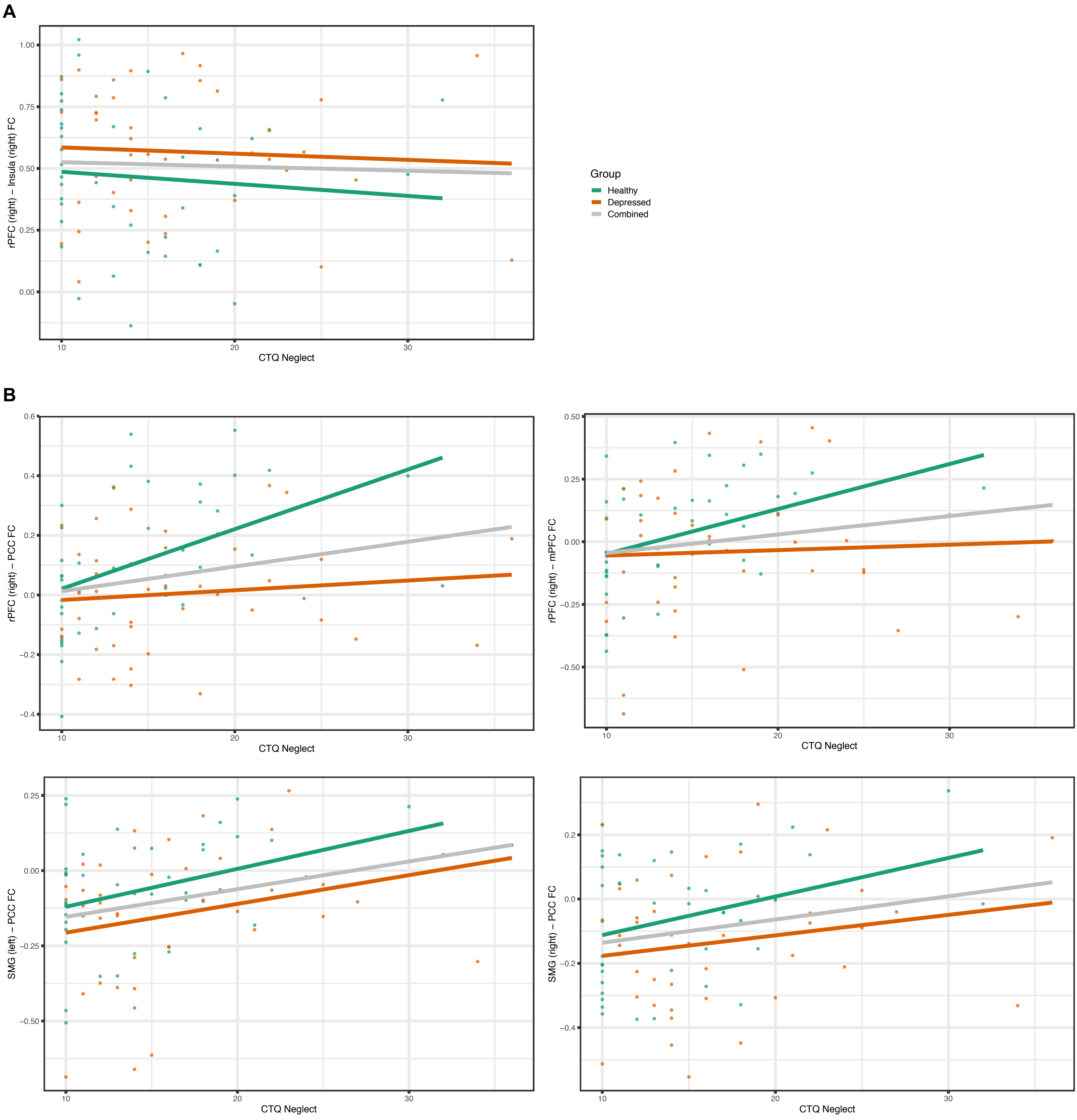

Figure S3. Associations between CTQ neglect and ROI-to-ROI functional connectivity (FC) for the depressed group, the healthy group, and combined, sorted by within- and between-network FC. A. Association between CTQ neglect and FC within the Salience Network. B. Associations between CTQ neglect and FC of the Salience with the Default Mode Network. ROI = region of interest, rPFC = rostral prefrontal cortex, mPFC = medial prefrontal cortex, SMG = supramarginal gyrus, PCC = precuneus cortex.

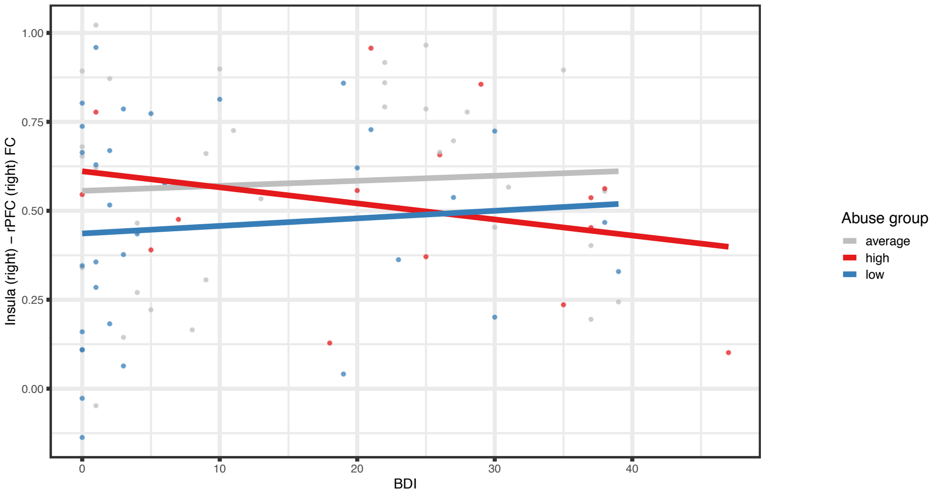

Figure S4. Moderation effect of CTQ abuse on the association between BDI-II and functional connectivity (FC) of the right rostral prefrontal cortex (rPFC) with the right Insula.
